# Immune Checkpoint Blockade for Metastatic Uveal Melanoma: Re-Induction following Resistance or Toxicity

**DOI:** 10.3390/cancers14030518

**Published:** 2022-01-20

**Authors:** Elias A. T. Koch, Anne Petzold, Anja Wessely, Edgar Dippel, Anja Gesierich, Ralf Gutzmer, Jessica C. Hassel, Sebastian Haferkamp, Katharina C. Kähler, Harald Knorr, Nicole Kreuzberg, Ulrike Leiter, Carmen Loquai, Friedegund Meier, Markus Meissner, Peter Mohr, Claudia Pföhler, Farnaz Rahimi, Dirk Schadendorf, Beatrice Schell, Max Schlaak, Patrick Terheyden, Kai-Martin Thoms, Beatrice Schuler-Thurner, Selma Ugurel, Jens Ulrich, Jochen Utikal, Michael Weichenthal, Fabian Ziller, Carola Berking, Markus V. Heppt, on behalf of the German Dermatologic Cooperative Oncology Group (DeCOG, Committee Ocular Melanoma)

**Affiliations:** 1 Department of Dermatology, Universitätsklinikum Erlangen, Friedrich-Alexander-University Erlangen-Nürnberg (FAU), 91054 Erlangen, Germany; elias.koch@uk-erlangen.de (E.A.T.K.); Anne.Petzold@uk-erlangen.de (A.P.); Anja.Wessely@uk-erlangen.de (A.W.); Beatrice.Schuler-Thurner@uk-erlangen.de (B.S.-T.); Carola.Berking@uk-erlangen.de (C.B.); 2 Comprehensive Cancer Center Erlangen-European Metropolitan Area of Nuremberg (CCC ER-EMN), 91054 Erlangen, Germany; 3 Department of Dermatology, Ludwigshafen Medical Center, 67059 Ludwigshafen, Germany; dippele@klilu.de; 4 Department of Dermatology, University Hospital Würzburg, 97080 Würzburg, Germany; gesierich_a@ukw.de; 5 Skin Cancer Center Minden, Department of Dermatology, Mühlenkreiskliniken AöR, Ruhr University Bochum Campus Minden, 32423 Minden, Germany; Ralf.Gutzmer@ruhr-uni-bochum.de; 6 Skin Cancer Center, Department of Dermatology and National Center for Tumor Diseases (NCT), University Hospital Heidelberg, 69120 Heidelberg, Germany; Jessica.Hassel@med.uni-heidelberg.de; 7 Department of Dermatology, University Hospital Regensburg, 93053 Regensburg, Germany; sebastian.haferkamp@ukr.de; 8 Department of Dermatology, University Hospital Schleswig-Holstein, Campus Kiel, 24105 Kiel, Germany; kkaehler@dermatology.uni-kiel.de (K.C.K.); mweichenthal@dermatology.uni-kiel.de (M.W.); 9 Department of Ophthalmology, Universitätsklinikum Erlangen, Friedrich-Alexander-University Erlangen-Nürnberg (FAU), 91054 Erlangen, Germany; Harald.Knorr@uk-erlangen.de; 10 Department of Dermatology and Venereology, Skin Cancer Center, Center of Integrated Oncology (CIO) Köln Bonn, University Hospital of Cologne, 50937 Cologne, Germany; nicole.kreuzberg@uk-koeln.de; 11 Department of Dermatology, Center for Dermatooncology, University Hospital Tübingen, 72056 Tübingen, Germany; ulrike.leiter@med.uni-tuebingen.de; 12 Department of Dermatology, University Medical Center Mainz, 55131 Mainz, Germany; carmen.loquai@unimedizin-mainz.de; 13 Skin Cancer Center, University Cancer Center Dresden and National Center for Tumor Diseases & Department of Dermatology, University Hospital Carl Gustav Carus, 01307 Dresden, Germany; Friedegund.Meier@uniklinikum-dresden.de; 14 Department of Dermatology, Venereology and Allergology, Goethe University, 60590 Frankfurt am Main, Germany; markus.meissner@kgu.de; 15 Department of Dermatology, Elbeklinikum, 21614 Buxtehude, Germany; peter.mohr@elbekliniken.de; 16 Department of Dermatology, Saarland University Medical School, 66421 Homburg, Saar, Germany; claudia.pfoehler@uks.eu; 17 Department of Dermatology and Allergy, Munich University Hospital (LMU), 81377 Munich, Germany; Farnaz.Rahimi@med.uni-muenchen.de; 18 Department of Dermatology, University Hospital Essen, University Duisburg-Essen, 45147 Essen, Germany; Dirk.Schadendorf@uk-essen.de (D.S.); Selma.Ugurel@uk-essen.de (S.U.); 19 German Cancer Consortium, Partner Site Essen, 45147 Essen, Germany; 20 Department of Dermatology, SRH Wald-Klinikum Gera, 07548 Gera, Germany; Beatrice.Schell@srh.de; 21 Charité—Universitätsmedizin Berlin, Corporate Member of Freie Universität Berlin, Humboldt-Universität zu Berlin and Berlin Institute of Health, Department of Dermatology, Venerology and Allergology, 10117 Berlin, Germany; max.schlaak@charite.de; 22 Department of Dermatology, University of Lübeck, 23562 Lübeck, Germany; patrick.terheyden@uksh.de; 23 Department of Dermatology, University Medical Center Goettingen, 37075 Goettingen, Germany; kai.thoms@med.uni-goettingen.de; 24 Department of Dermatology, Harzklinikum Dorothea Christiane Erxleben, 06484 Quedlinburg, Germany; jens.ulrich@harzklinikum.com; 25 Skin Cancer Unit, German Cancer Research Center (DKFZ) and Department of Dermatology, Venereology and Allergology, University Medical Center Mannheim, Ruprecht-Karl University of Heidelberg, 68167 Mannheim, Germany; Jochen.Utikal@umm.de; 26 Department of Dermatology, DRK Krankenhaus Rabenstein, 09117 Chemnitz, Germany; Ziller.Fabian@drk-khs.de

**Keywords:** uveal melanoma, immune checkpoint blockade, PD-1, CTLA-4, re-induction, treatment resistance, toxicity

## Abstract

**Simple Summary:**

The era of immune checkpoint blockade (ICB) with nivolumab and pembrolizumab (anti-PD-1) alone or in combination with ipilimumab (anti-CTLA-4) has led to prolonged survival in patients with cutaneous melanoma (CM). However, the response to ICB is low in patients with uveal melanoma (UM). This retrospective multicenter study examines the effectiveness of re-induction with ICB in patients with metastatic UM. A re-induction was recorded when ICB treatment was initiated a second time after a first ICB treatment was discontinued due to resistance or toxicity. We compared two cohorts (re-induction of ICB vs. once-only ICB) and present evidence for the clinical activity of a re-induction with ICB in a small subgroup of patients.

**Abstract:**

Re-induction with immune checkpoint blockade (ICB) needs to be considered in many patients with uveal melanoma (UM) due to limited systemic treatment options. Here, we provide hitherto the first analysis of ICB re-induction in UM. A total of 177 patients with metastatic UM treated with ICB were included from German skin cancer centers and the German national skin cancer registry (ADOReg). To investigate the impact of ICB re-induction, two cohorts were compared: patients who received at least one ICB re-induction (cohort A, *n* = 52) versus those who received only one treatment line of ICB (cohort B, *n* = 125). In cohort A, a transient benefit of overall survival (OS) was observed at 6 and 12 months after the treatment start of ICB. There was no significant difference in OS between both groups (*p* = 0.1) with a median OS of 16.2 months (cohort A, 95% CI: 11.1–23.8) versus 9.4 months (cohort B, 95% CI: 6.1–14.9). Patients receiving re-induction of ICB (cohort A) had similar response rates compared to those receiving ICB once. Re-induction of ICB may yield a clinical benefit for a small subgroup of patients even after resistance or development of toxicities.

## 1. Introduction

Uveal melanoma (UM) is the most common intraocular primary malignancy in adults and derives from uveal or ciliary body melanocytes [1]. This aggressive tumor metastasizes in around 50% of patients, which spreads predominantly to the liver [2]. The clinical course strongly depends on the genetic background of the primary tumor. Loss of heterozygosity for genes located on chromosome 3, duplication of genes on 8q, and loss of BAP1 are associated with poor prognosis [3]. The era of immune checkpoint blockade (ICB) with nivolumab and pembrolizumab (anti-PD-1) alone or in combination with ipilimumab (anti-CTLA-4) has led to significant survival benefits in patients with cutaneous melanoma (CM). However, the response rates to ICB in UM were consistently low, and potentially severe immune-related side effects may occur [4,5,6]. The objective response rate (ORR) of combined ICB lies within the range of 11.5–18% in two prospective phase II trials [7,8,9]. The bispecific molecule tebentafusp (IMCgp100) was recently approved by the FDA in the United States for patients suffering from advanced UM. It consists of a soluble T cell receptor binding to the gp100 peptide that is fused to an anti-CD3 single-chain variable fragment [10] and demonstrated a significant improvement in overall survival (OS) compared to patients receiving investigator’s choice as first-line treatment (21.7 vs. 16.0 months; *p* < 0.0001; stratified HR 0.51) [11]. However, the use of tebentafusp is restricted to patients exhibiting a specific HLA subtype (HLA-A02:01) which is found in only 45–50% of Caucasian populations. To this end, further treatment options are urgently needed and a re-induction of ICB is considered in many cases following resistance or toxicity to avoid chemotherapy in later treatment lines. In CM, a re-induction with anti-PD-1 with or without anti-CTLA-4 antibodies following acquired resistance to ICB has proven clinical activity in a subset of patients [12]. In this study, we investigated the value of ICB re-induction in a large nationwide collective of patients with metastatic UM.

## 2. Materials and Methods

### 2.1. Patient Population and Study Design

We performed a retrospective multicenter explorative analysis. Patients with metastatic UM receiving any type of ICB (ipilimumab, nivolumab, pembrolizumab, ipilimumab plus anti-PD-1 antibodies (referred to as combined ICB)) were eligible. A total of 177 patients were included and stratified into two cohorts. Cohort A comprised patients who underwent re-induction with ICB (*n* = 52, cohort A) while cohort B included those with only one treatment course of ICB (*n* = 125, cohort B). A re-induction was recorded when ICB treatment was initiated a second time after the first treatment with ICB was discontinued due to primary or acquired resistance or toxicity. For cohort A, response rates were analyzed both for the re-induction with ICB (A2) and the first treatment course with ICB (A1) as an additional intra-cohort control. In contrast, one best response to ICB was available and recorded for cohort B (B).

Clinical data and the treatment outcomes of interest were extracted from the original patient records from 15 German skin cancer centers (Erlangen *n* = 57, München *n* = 17, Tübingen *n* = 16, Mainz *n* = 7, Kiel *n* = 5, Mannheim *n* = 5, Heidelberg *n* = 4, Köln *n* = 3, Dresden *n* = 2 Homburg *n* = 2, Lübeck *n* = 2, Ludwigshafen *n* = 2, Essen *n* = 1, Frankfurt *n* = 1, Göttingen *n* = 1, Würzburg *n* = 1), as well as from the prospective multicentric skin cancer registry ADOReg of the German Dermatologic Cooperative Oncology Group (DeCOG) (*n* = 51). The ADOReg collects data for high-quality real-world evidence studies; all ADOReg patient IDs included in this study were checked for duplicates. The data were collected and merged into a central database before analysis. This study was approved by the scientific board of the ADOReg registry, the institutional review board of the medical faculty of the Munich University Hospital (approval number 413-16 UE), and it was conducted following the principles of the Helsinki Declaration in its current version.

### 2.2. Data Collection and Treatment Outcomes

The recorded clinical data at baseline comprised demographics with sex, age, number of organ systems affected by metastasis, and date of death or last documented patient contact. At the date of ICB initiation, the Eastern Cooperative Oncology Group (ECOG) performance status and serum lactate dehydrogenase (LDH) levels were collected from patient charts and analyzed for their prognostic values. Regarding the treatment, we recorded the number and type of therapies, ICB start and end dates, dates of progression on ICB, the best response to ICB (based on the RECIST criteria version 1.1), assessment of the frequency and adverse event (AE) assessment with grading based on the CTCAE criteria (version 5), and if the patients received radiation or liver-directed treatments. We summarized any metastases besides liver, bone, pulmonary, CNS, lymph node, connective tissue, and skin metastases as a category “other metastases”.

OS was calculated as the time from the date of treatment initiation of ICB until melanoma-specific or treatment-related death. Progression-free survival (PFS) was determined as the time from treatment start of ICB until disease progression confirmed by radiologic imaging or until clinically evident (if radiologic imaging was lacking because of rapid decline in clinical condition). Complete (CR) and partial (PR) responses were summarized as best ORR. Time-to-event analyses were calculated where death or disease progression were considered as events. If neither occurred or if patients were lost to follow-up, the date of the last documented presentation was used as a censored observation.

### 2.3. Statistical Analyses

The survival and progression probabilities were calculated with the Kaplan–Meier method. Log-rank tests were performed to compare the survival and progression probabilities of both cohorts. Furthermore, χ^2^ tests and t-tests were conducted (i) to test the comparability of the two cohorts, i.e., concerning possible different baseline characteristics and (ii) to compare the response to ICB between both cohorts. In all cases, two-tailed *p*-values were calculated and considered significant with values *p* < 0.05. Patients with missing values for a given variable were excluded. No imputation of missing data was performed. We refrained from using a propensity score due to the small sample size of this study, the different sizes of both cohorts, and the high number of missing values.

## 3. Results

### 3.1. Baseline Patient Characteristics

A total of 177 patients with metastatic UM were eligible and included; 47.5% of the patients had an ECOG status of 0 (*n* = 84), the serum LDH was elevated in 50.8% of cases (*n* = 90) at baseline. Both parameters were evenly distributed among both cohorts (48.1% vs. 47.2% and 40.4% vs. 55.2%, respectively). The patients had predominantly metastases to the liver (92.7%), lung (49.2%), bone (28.2%), lymph node (22.6%), CNS (13%), skin (13.6%), connective tissue (5.1%), and in 28.2% “other metastases”. Other baseline characteristics are listed in detail in Table 1. In cohort A, the first induction treatment with ICB was discontinued due to primary or acquired resistance and toxicity in 57.7% (*n* = 30) and 25% (*n* = 13), respectively. In 17.3% (*n* = 9), the reason for treatment discontinuation was unknown.

### 3.2. Response Rates to ICB

Combined ICB was applied in 119 patients (67.2%; cohort A 61.5% vs. cohort B 69.6%), while 5 patients received ipilimumab monotherapy (2.8%; cohort A 3.8% vs. cohort B 2.4%). PD-1 inhibitors were given as monotherapy in 53 patients (29.9%; cohort A 34.6% vs. cohort B 28%). The best ORR to any ICB in the entire population was 11.2% (17/152); 2% (3/152) of the patients achieved a CR, while 9.2% (14/152) had a PR.

The ORR to all ICB treatments was 10% (5/50) in cohort A (A2) vs. 11.8% (12/102) in cohort B (B) vs. 7.5% (4/52) in cohort A with ICB as the first induction treatment (A1), with no significant differences between the response rates (A2 vs. B: *p* = 0.95; A2 vs. A1: *p* = 0.95). Details of the patterns of response to ICB are summarized in Table 2. In cohort A after re-induction with ICB, two patients achieved a PR with anti-PD-1 monotherapy and combined ICB, respectively; one patient achieved a CR with combined ICB (Table 2, A2). In cohort A, the ORR to A2 was 11.1% (2/18), 50% (1/2), and 9.4% (3/32) for PD-1 inhibitor monotherapy, anti-CTLA-4 monotherapy, and combined ICB, respectively.

In total, 24/52 (46.2%) of the patients in cohort A showed PD to ICB treatment either in the first induction or re-induction. In cohort A, 63.5% (33/52) showed PD to the first induction with ICB. Of those, 6.1% (2/33) achieved a PR and 9.1% (3/33) SD to re-induction with ICB, resulting in an ORR of 6% and a DCR of 15.1% in this subgroup (Table 3). Among the 4 patients with a response to the first ICB induction, 2 (50%) showed PD, 1 (25%) PR, and another one (25%) CR. In cohort A, the ORR to A2 after discontinuation of the first ICB due to toxicity was higher than after discontinuation of the first ICB due to resistance, yet without significance (15.4% vs. 0%; *p* = 0.086).

### 3.3. Survival Data

**Figure 1:**
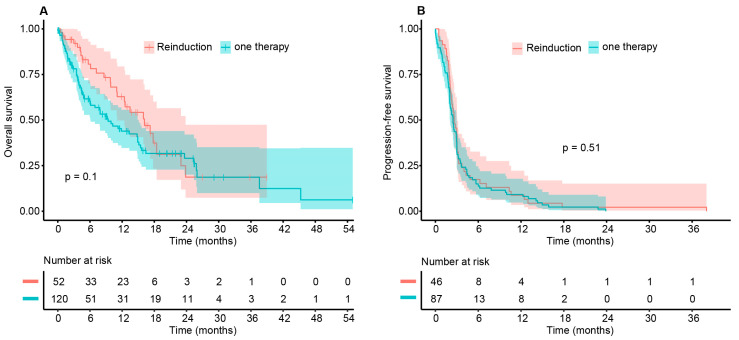
Kaplan–Meier estimates of the patient population for (**A**) OS and (**B**) PFS to ICB comparing cohort A (re-induction, red) vs. B (ICB one time, turquoise). Although there was no significant difference in OS and PFS (*p* = 0.1 and *p* = 0.51, respectively), the median survival differed considerably: cohort A 16.2 months (95%-CI: 11.1–23.8) vs. cohort B 9.4 months (95%-CI: 6.1–14.9). In contrast, the median PFS was equal in both cohorts: cohort A 2.6 months (95%-CI: 2.2–3.1) vs. cohort B 2.5 months (95%-CI 2.0–3.0). OS was not assessable for 5 patients of cohort B, PFS was not assessable for 6 patients of cohort A and 38 patients of cohort B.

The entire cohort showed a median OS of 11.9 months (95% CI 9.4–15.6) and a median PFS of 2.5 months (95% CI 2.2–3) to any ICB. There was no statistical difference in OS (*p* = 0.1) and PFS (*p* = 0.51) between both cohorts (Figure 1). Notably, a statistical difference in OS was observed after 6 and 12 months, which disappeared 18 and 24 months after the treatment start of ICB (Table 4).

### 3.4. Adverse Events (AE)

A total of 141 AE were reported in 74/177 (41.8%) patients. AE were observed in 24/52 (46.2%) patients in cohort A vs. in 50/125 (40%) patients in cohort B (*p* = 0.55). Severe AE (grade 3–5 according to CTCAE) occurred in 44/177 (24.8%) patients, with no difference between both cohorts (*p* = 0.82; Table 5).

The treatment was discontinued in 32 patients due to unacceptable toxicity. The most common AE were colitis (*n* = 26), hepatitis (*n* = 20), cutaneous toxicity (*n* = 12), thyroiditis (*n* = 10), hypophysitis (*n* = 8), myalgia with myositis (*n* = 6), and pancreatitis (*n* = 5). Interestingly, the toxicity to re-induction was significantly higher in those patients who experienced discontinuation to the first ICB due to toxicity than due to resistance (Table 6).

## 4. Discussion

Here, we present a retrospective, real-world, large cohort study of 177 patients with metastatic UM who were treated with ICB. This study aimed to investigate if re-induction with ICB is feasible under consideration of the dismal prognosis of metastatic UM, the short treatment window in stage IV, and the few ineffective therapy options available in this disease. The question of whether or not ICB should be re-induced is commonly asked in daily practice due to limited treatment options for UM. However, no data on this specific issue have been published to the best of our knowledge. ICB re-induction needs to be outweighed carefully due to the high treatment-related toxicities and costs.

We detected a median OS of 11.9 months (95% CI 9.4–15.6) and a median PFS of 2.5 months (95% CI 2.2–3) to any ICB for the entire cohort. The OS of 11.9 months is lower compared to previous retrospective studies, in which OS was 16 and 22.3 months in a real-world setting when treatment with ICB was available [4,5]. These data suggested that the prognosis of patients with advanced UM has improved due to the availability and more frequent use of ICB [4,5,9,13]. The lower OS in this collective is probably due to a relatively delayed treatment start, in particular in cohort A as the re-induction with ICB preferably occurred in later treatment lines. Defining the cohorts by re-induction with ICB itself makes an inherent selection for patients with longer survival times and a more favorable prognosis. Therefore, we also compared the response to re-induction (A2) to the first ICB treatment within cohort A (A1) yielding an additional control within cohort A, as was performed in previous studies investigating the value of reduction or re-challenge [14]. Nevertheless, OS should be interpreted with great caution due to this issue.

The PFS was equally poor in both cohorts. Validated prognostic parameters such as ECOG performance status or elevated serum levels of LDH were evenly distributed in both groups. However, many values were missing. The median number of affected organ systems was higher in cohort A, which was previously a favorable prognostic factor associated with long-term survival and treatment response to ICB [4]. This imbalance may act as a confounding factor regarding OS. Furthermore, the disease course in cohort A may inherently be less aggressive than in cohort B with more treatment lines being applied to the patients regardless of the treatment response. There was only a slight and transient OS benefit at 6 and 12 months after the treatment start of ICB between both cohorts which disappeared thereafter. Thus, it remains uncertain if the re-induction of ICB resulted in a true survival benefit.

In cohort A, two distinct response rates were calculated either to the first ICB induction (A1) or to re-induction with ICB (A2). The ORR A1 to any ICB (anti-PD-1 ± anti-CTLA-4) was 7.5% and the DCR was 34%. After the discontinuation due to resistance or toxicity, the re-induction with ICB yielded a response A2 in 10% and a DCR of 20%. These values were numerically lower but not significantly different from the ORR and DCR in cohort B (11.8% and 32.4%, respectively) and the first response A1 in cohort A. These data indicate that re-induction with ICB may offer clinical benefit in a rather small subset of patients, in particular, if the first ICB treatment line was discontinued due to toxicity, and represents a reasonable option to avoid cytoreductive chemotherapy in these patients. In contrast, the number of patients in cohort A achieving SD to the first ICB induction was higher compared to re-induction with ICB and showed a strong statistical trend (34% vs. 20%, *p* = 0.057). UM is rapidly progressing in later disease stages with a poor prognosis in studies before the ICB era [15,16]. Thus, a DCR of 20% in later treatment lines may still be regarded as a treatment success although a considerable portion of patients did not respond to re-induction with ICB in this study. Among 33 patients in cohort A who showed PD to the first ICB induction, 2 (6%) achieved a PR and 3 (9%) disease stabilization upon ICB re-induction. In contrast, 2 of the 4 patients showing a PR to first ICB induction experienced PD to ICB re-induction. These data highlight that both clinical benefit and disease progression may occur regardless of the previous response to the first ICB induction and stress that the decision to re-expose patients with UM to ICB needs to be made on a case-by-case basis.

While re-induction of ICB has not been investigated in metastatic UM to the authors’ knowledge, re-induction of ICB was performed in a panel of other cancer entities including CM [17]. In one retrospective multicenter study, patients with advanced CM who initially achieved at least a sustained SD to combined ICB and then progressed were re-inducted with ipilimumab alone or in combination with anti-PD-1, yielding an ORR of 26% (12/47) and a DCR of 45% (21/47) [12]. In this collective, severe toxicities were observed in 38% which is higher than in our population. In another small case series, eight patients were re-exposed to anti-PD-1 as monotherapy. One patient showed a PR, while three patients experienced SD resulting in a DCR of 50% [18]. However, the sample was small and no combined ICB of ipilimumab plus anti-PD-1 was investigated in this study. Similar results were observed in eight patients suffering from CM for the re-induction with nivolumab in a Japanese study [19]. Although the response rates for CM were, as expected, higher than reported in our study for UM, the patterns of response to re-induction are similar to the results of this dataset and highlight that re-induction represents an option for selected patients. If the general lack of immune response in UM is due to certain genetic alterations, to the low tumor mutational burden, to a low expression of neoantigens, or to the immune-suppressive tumor microenvironment needs to be further investigated, especially to identify subgroups of patients who can benefit from ICB either as first treatment or re-induction [20,21,22,23]. In Merkel cell carcinoma, re-induction with ICB revealed an ORR of 50% (4/8 patients) to anti-PD-1 therapy upon primary tumor progression [24]. However, prospective cohort studies are needed to validate these observations in larger cohorts.

In this population, AE occurred in 41.8% of patients with no difference between cohorts A and B, demonstrating that the re-induction was not associated with a higher risk of immune-related toxicities. The rates of AE and severe AE were evenly distributed among both cohorts, suggesting that re-induction with ICB was not accompanied by additional or unforeseen safety concerns. The rate of severe AE of 24.8% is in line but on the lower range of previously published studies where immune-related grade 3/4 toxicities occurred in about 30–60% of patients upon combined ICB [7,8,9,25]. Notably, the toxicity of re-induction was significantly higher in patients who experienced discontinuation to the first ICB due to toxicity than due to resistance. Thus, safety needs to be carefully evaluated in this subgroup.

The major limitation of this study is its retrospective design, the small numbers of responders to ICB, the high number of missing values, and the inherent selection of patients with a favorable prognosis by defining the cohorts for re-induction with ICB. Due to the small sample size, missing values, and the size asymmetry of both cohorts we refrained from using a propensity score which could have helped to better control for confounding variables in this study. The quality and completeness of the data, in particular the reporting of AE, is highly dependent on the participating cancer centers. Thus, we cannot exclude that AE were underreported in this study. Furthermore, the response rates A1 and A2 were considered as independent events for the sake of analysis although both events were reported in the same patient cohort. To make the comparison of the re-induction more robust, cohort B was compared as an independent collective where ICB was given once only without a re-exposure. This imbalance of the treatment courses between both cohorts may introduce bias and we cannot exclude that cohort A was prognostically favorable per se regardless of any treatment effects.

## 5. Conclusions

Nevertheless, these data demonstrate that re-induction with ICB was feasible, did not induce additional toxicity, and may offer clinical benefit in a small subset of patients with advanced UM when no other treatment options are left.

## Figures and Tables

**Table 1:** Characteristics of the study population. Abbreviations: NA = not available, ICB = immune checkpoint blockade.

		Total	Cohort A	Cohort B	A vs. B
Sex	Women	89 (50.3%)	27 (48.1%)	62 (49.6%)	*p* = 0.9
	Men	88 (49.7%)	25 (51.9%)	63 (50.4%)	
Age	Median in years (range)	66.2 (17.7–87.6)	64.2 (31.7–85.7)	67.0 (17.7–87.6)	*p* = 0.3
LDH	Not elevated	40 (22.6%)	14 (26.9%)	26 (20.8%)	*p* = 0.1
	Elevated	90 (50.8%)	21 (40.4%)	69 (55.2%)	
	NA	47 (26.6%)	17 (32.7%)	30 (24.0%)	
ECOG	ECOG 0	84 (47.5%)	25 (48.1%)	59 (47.2%)	*p* = 0.22
	ECOG 1	19 (10.7%)	4 (7.7%)	15 (12.0%)	
	ECOG 2	4 (2.3%)	1 (1.9%)	3 (2.4%)	
	ECOG 3	2 (1.1%)	0 (0.0%)	2 (1.6%)	
	ECOG 4	0 (0.0%)	0 (0.0%)	0 (0.0%)	
	ECOG 5	0 (0.0%)	0 (0.0%)	0 (0.0%)	
	NA	68 (38.4%)	22 (42.3%)	46 (36.8%)	
Number of affected organ systems	Median (range)	2 (1–8)	3 (1–7)	2 (1–8)	*p* = 0.013
Affected organ systems	Liver	164 (92.7%)	50 (96.2%)	114 (91.2%)	
	Pulmonary	87 (49.2%)	27 (51.9%)	60 (48.0%)	
	Bone	50 (28.2%)	19 (36.5%)	31 (24.8%)	
	CNS	23 (13.0%)	10 (19.2%)	13 (10.4%)	
	Lymph node	40 (22.6%)	15 (28.9%)	25 (20.0%)	
	Connective tissue	9 (5.1%)	4 (7.7%)	5 (4.0%)	
	Skin	24 (13.6%)	12 (23.1%)	12 (9.6%)	
	Disseminated	10 (5.6%)	2 (3.8%)	8 (6.4%)	
	Other	50 (28.2%)	20 (38.5%)	30 (24.0%)	
Treatments other than ICB	Chemotherapy	46 (25.9%)	28 (53.8%)	18 (14.4%)	*p* < 0.001
	MEK inhibitor	13 (73.4%)	4 (7.6%)	9 (7.2%)	*p* > 0.999
	Sorafenib	22 (12.4%)	9 (17.3%)	13 (10.4%)	*p* = 0.308
ICB regimen	Any	177 (100%)	52 (100.0%)	125 (100%)	
	Anti-PD-1 monotherapy (pembrolizumab, nivolumab)	53 (29.9%)	18 (34.6%)	35 (28.0%)	*p* = 0.487
	Anti-CTLA-4 monotherapy (ipilimumab)	5 (2.8%)	2 (3.8%)	3 (2.4%)	*p* = 0.975
	Combined ICB	119 (67.2%)	32 (61.5%)	87 (69.6%)	*p* = 0.387

**Table 2:** Response rates to ICB. Abbreviations: CR = complete response, PR = partial response, SD = stable disease, PD = progressive disease, ORR = objective response rate, DCR = disease control rate. No response data was assessable for 25 patients in total (2 patients in cohort A2 and 23 patients in cohort B). Patients with mixed response are not listed (3 patients of cohort A2, 2 patients of cohort A1, 5 patients of cohort B).

	Total	Cohort A		Cohort B	Test		
		ICB First Induction (A1)	ICB Re-Induction (A2)		A1 vs. A2	A1 vs. B	A2 vs. B
CR	3/152 = 2.0%	0/52 = 0.0%	1/50 = 2.0%	2/102 = 2.0%	*p* = 0.98	*p* = 0.79	*p* > 0.999
PR	14/152 = 9.2%	4/52 = 7.7%	4/50 = 8.0%	10/102 = 9.8%	*p* > 0.999	*p* = 0.89	*p* = 0.94
SD	26/152 = 17.1%	13/52 = 25.0%	5/50 = 10.0%	21/102 = 20.6%	*p* = 0.084	*p* = 0.68	*p* = 0.16
PD	101/152 = 66.4%	33/52 = 63.5%	37/50 = 74.0%	64/102 = 62.7%	*p* = 0.35	*p* > 0.999	*p* = 0.23
ORR	17/152 = 11.2%	4/52 = 7.7%	5/50 = 10.0%	12/102 = 11.8%	*p* = 0.95	*p* = 0.61	*p* = 0.95
DCR	43/152 = 28.3%	18/52 = 34.6%	10/50 = 20.0%	33/102 = 32.4%	*p* = 0.15	*p* = 0.91	*p* = 0.16

**Table 3:** Response rates to ICB in cohort A. Abbreviations: CR = complete response, PR = partial response, SD = stable disease, PD = progressive disease. Patients with a mixed response and no assessable response data are not listed. Two patients had a mixed response to the first ICB, both showing PD to ICB re-induction, and two patients had a mixed response to ICB re-induction after PD to the first ICB. Additionally, two patients were not assessable for re-induction after PD to the first ICB.

Response to First ICB Induction	PD to ICB Re-Induction	SD to ICB Re-Induction	PR to ICB Re-Induction	CR to ICB Re-Induction
PD (*n* = 33/52)	24 (72.7%)	3 (9%)	2 (6%)	0 (0.0%)
SD (*n* = 13/52)	9 (69.2%)	2 (15.3%)	1 (7.6%)	0 (0.0%)
PR (*n* = 4/52)	2 (50%)	0 (0.0%)	1 (25%)	1 (25%)

**Table 4:** Survival probabilities after 6, 12, 18, and 24 month landmarks. Abbreviations: OS = overall survival.

Time in Months	OS (95% CI) Cohort A	OS (95% CI) Cohort B	Difference (95% CI)
6	0.806 (0.6997–0.929)	0.604 (0.516–0.708)	0.202 (0.139–0.265)
12	0.628 (0.4974–0.794)	0.439 (0.347–0.556)	0.189 (0.085–0.293)
18	0.374 (0.2325–0.601)	0.317 (0.228–0.440)	0.057 (−0.087–0.201)
24	0.187 (0.0737–0.474)	0.290 (0.200–0.420)	−0.103 (−0.240–0.033)

**Table 5:** Occurrence of adverse events. Abbreviations: AE = adverse events.

	Total	Cohort A1	Cohort A2	Cohort B	*p*-Value (A1 vs. B)
Number of patients with any AE	74/177 (41.8%)	24/52 (46.2%)	21/52 (40.4%)	50/125 (40.0%)	*p* = 0.55
Number of patients with severe AE	44/177 (24.8%)	14/52 (26.9%)	10/52 (19.1%)	30/125 (24.0%)	*p* = 0.82

**Table 6:** Occurrence of adverse events. Abbreviations: AE = adverse events.

Toxicity of Re-Induction	Re-Induction after Resistance to First ICB (*n* = 30)	Re-Induction after Toxicity to First ICB (*n* = 13)	*p*-Value
Number of patients with any AE	6 (20.0%)	13 (100.0%)	*p* < 0.0001
Number of patients with severe AE	3 (10.0%)	5 (38.5%)	*p* = 0.04

## Data Availability

Data are contained within the article.

## References

[1] Smit K.N., Jager M.J., de Klein A., Kili E. (2020). Uveal melanoma: Towards a molecular understanding. Prog. Retin. Eye Res..

[2] Collaborative Ocular Melanoma Study Group (2001). Assessment of metastatic disease status at death in 435 patients with large choroidal melanoma in the Collaborative Ocular Melanoma Study (COMS): COMS report no. 15. Arch. Ophthalmol..

[3] Harbour J.W., Onken M.D., Roberson E.D., Duan S., Cao L., Worley L.A., Council M.L., Matatall K.A., Helms C., Bowcock A.M. (2010). Frequent mutation of BAP1 in metastasizing uveal melanomas. Science.

[4] Koch E.A.T., Petzold A., Wessely A., Dippel E., Erdmann M., Heinzerling L., Hohberger B., Knorr H., Leiter U., Meier F. (2021). Clinical determinants of long-term survival in metastatic uveal melanoma. Cancer Immunol. Immunother..

[5] Koch E.A.T., Petzold A., Wessely A., Dippel E., Gesierich A., Gutzmer R., Hassel J.C., Haferkamp S., Hohberger B., Kahler K.C. (2021). Immune Checkpoint Blockade for Metastatic Uveal Melanoma: Patterns of Response and Survival According to the Presence of Hepatic and Extrahepatic Metastasis. Cancers.

[6] Koch E.A.T., Nickel F.T., Heinzerling L., Schulz Y.K., Berking C., Erdmann M. (2021). Immune Checkpoint Inhibitor-induced Bilateral Vestibulopathy. J. Immunother..

[7] Pelster M.S., Gruschkus S.K., Bassett R., Gombos D.S., Shephard M., Posada L., Glover M.S., Simien R., Diab A., Hwu P. (2020). Nivolumab and Ipilimumab in Metastatic Uveal Melanoma: Results From a Single-Arm Phase II Study. J. Clin. Oncol..

[8] Piulats J.M., Espinosa E., de la Cruz Merino L., Varela M., Alonso Carrion L., Martin-Algarra S., Lopez Castro R., Curiel T., Rodriguez-Abreu D., Redrado M. (2021). Nivolumab Plus Ipilimumab for Treatment-Naive Metastatic Uveal Melanoma: An Open-Label, Multicenter, Phase II Trial by the Spanish Multidisciplinary Melanoma Group (GEM-1402). J. Clin. Oncol..

[9] Heppt M.V., Amaral T., Kahler K.C., Heinzerling L., Hassel J.C., Meissner M., Kreuzberg N., Loquai C., Reinhardt L., Utikal J. (2019). Combined immune checkpoint blockade for metastatic uveal melanoma: A retrospective, multi-center study. J. Immunother. Cancer.

[10] Bossi G., Buisson S., Oates J., Jakobsen B.K., Hassan N.J. (2014). ImmTAC-redirected tumour cell killing induces and potentiates antigen cross-presentation by dendritic cells. Cancer Immunol. Immunother..

[11] Piperno-Neumann S., Hassel J.C., Rutkowski P., Baurain J.-F., Butler M.O., Schlaak M., Sullivan R.J., Ochsenreither S., Dummer R., Kirkwood J.M. (2021). Abstract CT002: Phase 3 randomized trial comparing tebentafusp with investigator’s choice in first line metastatic uveal melanoma. Cancer Res..

[12] Hepner A., Atkinson V.G., Larkin J., Burrell R.A., Carlino M.S., Johnson D.B., Zimmer L., Tsai K.K., Klein O., Lo S.N. (2021). Re-induction ipilimumab following acquired resistance to combination ipilimumab and anti-PD-1 therapy. Eur. J. Cancer.

[13] Wessely A., Steeb T., Erdmann M., Heinzerling L., Vera J., Schlaak M., Berking C., Heppt M.V. (2020). The Role of Immune Checkpoint Blockade in Uveal Melanoma. Int. J. Mol. Sci..

[14] Tietze J.K., Forschner A., Loquai C., Mitzel-Rink H., Zimmer L., Meiss F., Rafei-Shamsabadi D., Utikal J., Bergmann M., Meier F. (2018). The efficacy of re-challenge with BRAF inhibitors after previous progression to BRAF inhibitors in melanoma: A retrospective multicenter study. Oncotarget.

[15] Khoja L., Atenafu E.G., Suciu S., Leyvraz S., Sato T., Marshall E., Keilholz U., Zimmer L., Patel S.P., Piperno-Neumann S. (2019). Meta-analysis in metastatic uveal melanoma to determine progression free and overall survival benchmarks: An international rare cancers initiative (IRCI) ocular melanoma study. Ann. Oncol..

[16] Rantala E.S., Hernberg M., Kivela T.T. (2019). Overall survival after treatment for metastatic uveal melanoma: A systematic review and meta-analysis. Melanoma. Res..

[17] Zaremba A., Eggermont A.M.M., Robert C., Dummer R., Ugurel S., Livingstone E., Ascierto P.A., Long G.V., Schadendorf D., Zimmer L. (2021). The concepts of rechallenge and retreatment with immune checkpoint blockade in melanoma patients. Eur. J. Cancer.

[18] Blasig H., Bender C., Hassel J.C., Eigentler T.K., Sachse M.M., Hiernickel J., Koop A., Satzger I., Gutzmer R. (2017). Reinduction of PD1-inhibitor therapy: First experience in eight patients with metastatic melanoma. Melanoma. Res..

[19] Nomura M., Otsuka A., Kondo T., Nagai H., Nonomura Y., Kaku Y., Matsumoto S., Muto M. (2017). Efficacy and safety of retreatment with nivolumab in metastatic melanoma patients previously treated with nivolumab. Cancer Chemother. Pharmacol..

[20] Hoefsmit E.P., Rozeman E.A., Van T.M., Dimitriadis P., Krijgsman O., Conway J.W., Pires da Silva I., van der Wal J.E., Ketelaars S.L.C., Bresser K. (2020). Comprehensive analysis of cutaneous and uveal melanoma liver metastases. J. Immunother. Cancer.

[21] Schumacher T.N., Scheper W., Kvistborg P. (2019). Cancer Neoantigens. Annu. Rev. Immunol..

[22] Hashimoto K., Nishimura S., Ito T., Akagi M. (2021). Characterization of PD-1/PD-L1 immune checkpoint expression in soft tissue sarcomas. Eur. J. Histochem..

[23] Hashimoto K., Nishimura S., Sakata N., Inoue M., Sawada A., Akagi M. (2021). Characterization of PD-1/PD-L1 immune checkpoint expression in the pathogenesis of musculoskeletal Langerhans cell histiocytosis: A retrospective study. Medicine.

[24] Stege H.M., Haist M., Schultheis S., Fleischer M.I., Mohr P., Ugurel S., Terheyden P., Thiem A., Kiecker F., Leiter U. (2021). Response durability after cessation of immune checkpoint inhibitors in patients with metastatic Merkel cell carcinoma: A retrospective multicenter DeCOG study. Cancer Immunol. Immunother..

[25] Najjar Y.G., Navrazhina K., Ding F., Bhatia R., Tsai K., Abbate K., Durden B., Eroglu Z., Bhatia S., Park S. (2020). Ipilimumab plus nivolumab for patients with metastatic uveal melanoma: A multicenter, retrospective study. J. Immunother. Cancer.

